# Effects of ex situ conservation on commensal bacteria of crocodile lizard and conservation implications

**DOI:** 10.1080/01652176.2025.2463704

**Published:** 2025-02-10

**Authors:** Haiying Jiang, Mei Lv, Tengfei He, Mujiao Xie, Zhiwen Zhao, Jiasong He, Shuyi Luo, Yide Guo, Jinping Chen

**Affiliations:** a Guangdong Key Laboratory of Animal Conservation and Resource Utilization, Institute of Zoology, Guangdong Academy of Sciences, Guangzhou, China; b Guangxi Daguishan Crocodile Lizard National Nature Reserve, Hezhou, China

**Keywords:** Conservation, captivity, community assembly, disease, environment, microbiome, *Shinisaurus crocodilurus*

## Abstract

Ex situ conservation is an important wildlife conservation strategy, but endangered wildlife in captivity often exhibit high disease rates. Commensal microorganisms are vital for homeostasis, immunity, and linked to diseases. This study analyzed the structure, assembly, variations of the symbiotic microbiota of the endangered crocodile lizard, and their relationship with environment, as well as the effects of captivity on them, to explore why captive reptiles face high dermatosis rates. Results showed that the reptile’s microbiota significantly differ from that of its habitat, demonstrating niche specificity. While species richness among organs showed no significant differences, microbial diversity varied considerably. Skin microbiota showed no site-specific clustering. The assembly of skin, oral, and intestinal bacterial communities was dominated by homogeneous selection. The gut and oral bacterial networks were resilient to disturbances, while the skin bacterial network was sensitive. Captivity primarily affected the skin microbiota, reducing its diversity and stability, thereby increasing disease risk, and these effects were not solely attributable to environmental changes. These findings suggested that skin microbial changes in captive reptiles may be responsible for their increased susceptibility to dermatosis in ex situ conservation. This study underscored the importance of understanding reptile-associated microbes for effective conservation strategies and offers potential solutions.

## Introduction

Biodiversity is crucial to our earth, and is increasingly threatened. Globally, 40.7% of amphibians, 25.4% of mammals, 13.6% of birds and 21.1% of reptiles are at risk of extinction (Cox et al. 2022). Ex-situ conservation is an effective complement to in-situ conservation, helping to protect and restore endangered wildlife by expanding their population through artificial breeding and reintroducing them back to natural habitats. While captivity provides refuge and ample food for endangered animals, it also subjects them to dietary changes, environmental stress, habitat homogenization, increased density, inbreeding, etc. These stressors are likely to increase disease susceptibility and exacerbate the detrimental impacts of disease outbreaks on population viability. In practice, it is often observed that endangered wildlife in captivity exhibit a tendency towards high disease rate. For instance, the prevalence of skin diseases among captive crocodile lizards can peak at 53.75%, significantly higher than that observed in wild populations (Jiang et al. 2022). Similarly, the prevalence of malaria parasites is greater in captive/semicaptive population (29 of 31) compared to wild populations (Wolfe et al. 2002). And the proportion of species that have been successfully recovered to date is small. According to the data from International Union for Conservation of Nature (IUCN), only 16 species of threatened animals have shown population increases in the last two years, while 1,473 species have continued to decline (IUCN 2024). Therefore, it is essential to assess the effect of captivity on threatened animals to improve conservation strategies.

Recent studies showed significant changes in the commensal microbial communities of some captive endangered animals (Dallas and Warne 2023, West et al. 2019). Commensal microorganisms are vital for the body homeostasis and immunity, and are associated with many diseases (Trevelline et al. 2019). Therefore, understanding the structure and assembly of the commensal microorganisms, as well as the effects of captivity is crucial for improvements in animal health and the captive breeding and reintroduction programs. While commensal microorganisms across body sites have been well studied in humans, such studies in non-human species remain relatively scarce (Ding et al. 2022, Kohl et al. 2017, Lutz et al. 2019, McKnight et al. 2020, Montoya-Ciriaco et al. 2023, Pereira and Clemente 2021, Roggenbuck et al. 2014, Smith et al. 2021, Zhao et al. 2022, Zou et al. 2023). Most microbial studies in non-human species have focused on the gut microbiome, with relatively limited understanding of microbiome in other body sites. We conducted a brief statistical analysis utilizing the Web of Science database (https://webofscience.clarivate.cn/), and found that among the articles investigating the microbiome of non-human vertebrates, a significant proportion of 77.7% concentrated on the gut microbiome, whereas only 8.6% were dedicated to the oral microbiome and 4.4% to the skin microbiome (Supplemental Table S1). In addition, the heterogeneity of the microbiome of the skin, the largest organ, cannot be represented by a single swab collected throughout the body or a single site.

Reptile species differ from mammals in physiology, behavior, and lifestyle. For example, most reptiles move with their bellies on the ground or just slightly off the ground, which puts them in more contact with their environment than other animals. Consequently, their microbial community structure and variation are likely distinct from those of mammals. In addition, different body sites provide diverse niches for microbial growth, and therefore they may also exhibit unique microbiota at specific body sites. Studying the body-wide microbiome of reptile is necessary to gain a comprehensive understanding of reptile microbiome and the effects of captivity on them. Furthermore, since most health problems in herpetology are in immunocompromised patients due to improper captive strategies (Mader 2006), a comprehensive understanding of the normal reptile microbiome across body sites facilitates clinical diagnosis and treatment.

The variation in animal-associated microbiome is influenced by intrinsic factors such as species, genetics, sex, age and lifestyle, and environmental factors like temperature, diet, surrounding microorganisms (Ross et al. 2019, Youngblut et al. 2019). These studies are generally conducted on a single site of microbial communities. To date, few studies have investigated the simultaneous effects of the same factor on microbiota in different body sites with different physiological functions, which is critical for predicting disease and managing host health in captive breeding and reintroduction programs.

The crocodile lizard (*Shinisaurus crocodilurus*) is a semiaquatic lizard native to China and Vietnam, and the sole living species in the family Shinisauridae, described as a living fossil reptile. This species is listed as endangered on the IUCN Red List, and is classified as one of the national first-class protected animals in China. The wild population of crocodile lizard is smaller than that of the well-known endangered giant panda, with an estimated total of 1,167-1,325 individuals (Jiang et al. 2022). Several nature reserves are currently engaged in the ex situ captive breeding and release program to save this lizard from endangerment. China has established two national nature reserves with crocodile lizards as the main objects of protection: the Guangxi Daguishan Crocodile Lizard National Nature Reserve and the Guangdong Luokeng Crocodile Lizard National Nature Reserve. However, like other captive animals, the crocodile lizards in ex situ captivity suffer from various diseases, especially skin diseases, one of the most common diseases in reptiles (Brady et al. 2016; Jiang et al. 2017, 2019, 2022). Our previous study found that skin diseases are associated with changes in skin microbiome, so here we further investigate whether captivity makes skin microbiome vulnerable (Jiang et al. 2022).

The semi-aquatic lifestyle of the crocodile lizard makes it particularly suitable for studying changes in the microbial community and environmental effects of reptiles that have close contact with their surroundings. In this study, the crocodile lizard was used as an example to analyze the effects of ex-situ conservation on commensal microbiota of different body sites in reptiles. This research will guide conservation projects aimed at reducing the impact of captivity on endangered animals.

## Materials and methods

### Ethics statement

This study was conducted in accordance with the guidelines of the Committee on the Ethics of Animal Experiments of the Institute of Zoology, Guangdong Academy of Sciences, following basic principles, and in accordance with the current laws on animal welfare and research in China.

### Sample collection

Samples were collected from Guangxi Daguishan and Guangdong Luokeng Crocodile Lizard National Nature Reserves in China. Given our previous finding that the skin microbiota of crocodile lizards are related to skin diseases (Jiang et al. 2022), all crocodile lizards sampled in this study were healthy adults to minimize the interference of diseases and age factors. Sterile swabs were used to collect the microbes from the skin, oral cavity and cloaca of the crocodile lizards. Specifically, the skin samples were divided into four parts: head, back, belly and paw (Supplemental Figure S1). Both wild and captive crocodile lizards were sampled at the same nature reserve. Additionally, back and belly samples from captive crocodile lizards in Guangxi Daguishan Crocodile Lizard National Nature Reserve were collected continuously for four months (end of June to end of September) to track the variation of skin microbiota. Five adult crocodile lizards were included in each group as biological replicates. To identify the microbes in habitat of crocodile lizards, water and soil samples from wild streams and feeding ponds were also collected, with three replicates in each site. For the water samples, the precipitate was collected using vacuum filtration with 0.22 μm filter membrane.

A total of 192 samples were collected, including 110 skin samples, 20 oral samples, 20 intestinal samples, 21 water samples, and 21 soil samples. All samples were stored in RNA-Be-Locker A (Sangon Biotech (Shanghai) Co., Ltd., China) and transported with liquid nitrogen to the lab for DNA extraction.

To measure the effect of habitat water quality on the microbial community, 8 water quality indicators were measured at each sampling site. These indicators included pH, water temperature (T), dissolved oxygen (DO), chemical oxygen demand (COD), total nitrogen (TN), total phosphorus (TP), permanganate index (I_Mn_), and ammonia nitrogen (NH3-N). The pH was measured using a PH Meter in Smart Sensor (PH809, China); water temperature and dissolved oxygen were detected using a dissolved oxygen analyzer in Smart Sensor (AR8010, China); and the other parameters were detected by portable water quality detector (RB-900F, Guangzhou Ruibin Technology Co., Ltd, China) according to the manufacturer’s instructions. Details of the methodology used were identical to our previous study (Xie et al. 2023).

### DNA extraction and sequencing

Total DNA from samples was extracted using TGuide S96 Magnetic Soil/Stool DNA Kit DP812 (TIANGEN Biotech (Beijing) Co., Ltd.). The V3-V4 hypervariable regions of the 16S rRNA gene were amplified using the primers F (5′-ACTCCTACGGGAGGCAGCA-3′) and R (5′-GGACTACHVGGGTWTCTAAT-3′). High-throughput sequencing library construction and sequencing were performed by Biomarker Technologies Corporation (China). Sequencing was conducted on the Illumina NovaSeq 6000 platform (250 bp paired-end reads).

### Sequence analysis

The raw reads were submitted to microbial diversity analysis platform on BMKCloud (http://www.biocloud.net/) for data analysis. After quality control, amplicon sequence variants (ASVs) were generated by Dada2 in QIIME2 (version 2020.6) (https://qiime2.org/). ASVs were annotated using SILVA Release 138 as reference database. To analyze the bacterial community data more accurately, only the ASVs annotated to the bacteria were retained. QIIME2 was used to determine the abundance of each species. For comparison among groups, the ASVs abundances were normalized based on the number of sequences in the sample with the lowest counts.

### Statistical analysis

Statistical analyses were performed and visualized by R software (version 4.2.3).

Alpha and beta diversity analysis were processed by QIIME2 (version 2020.6). Alpha diversity was showed by ACE and Shannon indexes, and beta diversity was measured by binary Jaccard, Bray-Curtis and weighted Unifrac distance matrices. For the comparison of alpha diversity, ANOVA test and Scheffe multiple comparison were performed to test the significance of differences among groups. Principal coordinate analysis (PCoA), non-metric multidimensional scaling analysis (NMDS), unweighted pair-group method with arithmetic means (UPGMA) clustering graphs were drawn to show beta diversity. For the comparison of beta diversity, PERMANOVA test was conducted to test the significance of difference among groups using “adonis2” function in the vegan package. The PERMDISP test was conducted to analyze the multivariate homogeneity of group dispersions using “betadisper” function in the vegan package.

Biomarker discovery between groups was conducted by the linear discriminatory analysis (LDA) effect size (LEfSe) (Segata et al. 2011), with parameters set to LDA > 4.0 and *p* < 0.05.

Biological phenotypes of bacteria (oxygen tolerance, biofilm formation, pathogenicity) were predicted by BugBase (https://bugbase.cs.umn.edu/index.html). Functional pathway predictions for bacteria were conducted using PICRUSt2 (Douglas et al. 2020).

Microbial community assembly processes were quantified using the neutral community model (NCM) and null model (Zhou and Ning 2017). The R script for the NCM analysis followed that of Chen et al. (2019). In the null model, a value of |βNTI| > 2 indicates deterministic processes, which could be divided into homogeneous selection (βNTI < −2) and heterogeneous selection (βNTI > +2). Otherwise, |βNTI| < 2 indicates stochastic processes, which could be divided into homogenizing dispersal (RC_bary_ < −0.95), dispersal limitation (RC_bary_ > +0.95), and drift (|RCbary| < 0.95, referred to as “undominated” processes in this study). The R script for the null model analysis was modified from that of Zhang et al. (2019).

Network analysis was used to explore the stability of microbial networks in response to disturbance (Wu et al. 2021). A bacterial co-occurrence network was constructed based on Spearman correlations among ASVs using the “network.pip” function in the ggClusterNet package (Wen et al. 2022). The ASV tables were filtered by top 500 relative abundance, and Spearman correlation results were filtered by the thresholds *r* > 0.75 and *p* < 0.05. Network stability was measured through natural connectivity changes against node removal using the “natural.con.microp” function in the ggClusterNet package (Wu et al. 2021; Wen et al. 2022).

The R package “SourceTracker” was used to estimate the sources of skin bacteria (Knights et al. 2011), with ASVs from skin samples as sink, and ASVs from gut, mouth, water, and soil samples as source. The Mantel test was used to analyze the correlation between the variation of microbiota and water quality indicators.

## Results

### Overview of data

A total of 29,417,871 raw reads were obtained from 192 samples. After quality control and assembly, 29,287,985 clean reads were generated, with at least 80,130 clean reads per sample. The rarefaction curves for each sample tended to flatten out, indicating sufficient sequencing depth in this study (Supplemental Figure S2). After normalization, each sample contained 77,732 sequences. Subsequently, clustering yielded 220,776 ASVs, with 76–4,806 per sample (median is 1,541). Of all the ASVs, 78.7% could be assigned to the family level and 49.7% to the genus level. These ASVs were distributed across 43 phyla, 124 classes, 434 orders, 1,104 families, 2,980 genera, and 5,070 species.

### Microbial composition and diversity vary among organs

To investigate differences in microbial communities among organs, and skin sites, we selected 144 samples collected simultaneously out of 192 sequenced samples.

**Figure 1.**
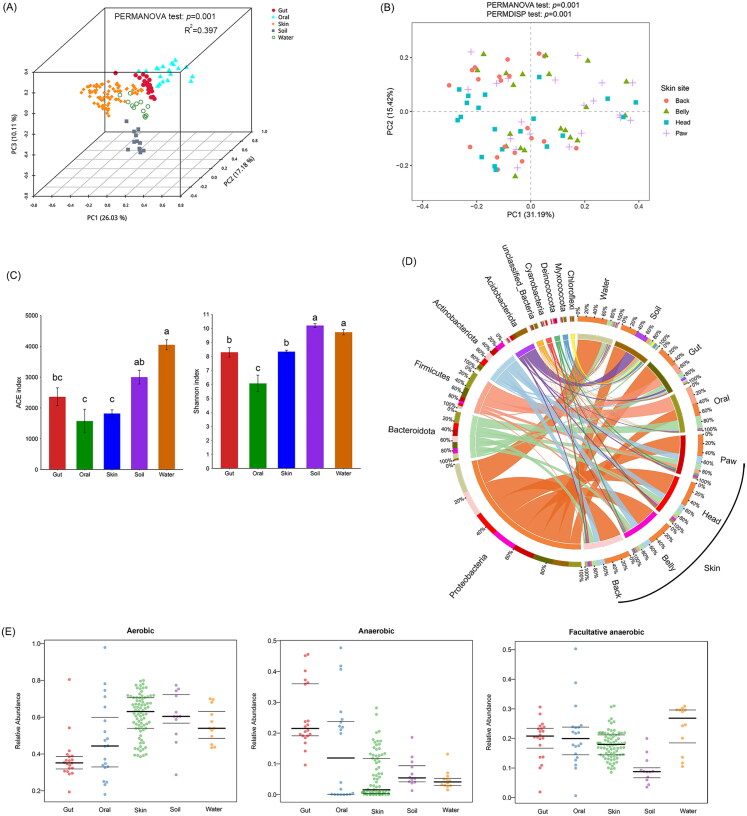
Distribution of bacterial communities of crocodile lizards and habitat environment. Beta diversity among organs (A) and skin sites (B) were showed using PCoA based on weighted Unifrac distance matrix. Numbers in the parenthesis on the axis labels indicate the percentage of variation explained by each PC. Differences in beta diversity among groups were detected by PERMANOVA. Analysis of the multivariate homogeneity of group dispersions was performed by PERMDISP test. Alpha diversity among organs was shown using ACE and Shannon indices (C). Differences in alpha diversity among groups were analyzed using ANOVA test and Scheffe’ multiple comparison. Data with different letters above the bars are significantly different (*p* < 0.05), and error bars indicate the standard deviation. Bacterial composition was showed at the phylum level highlighting the top 10 most abundant taxa (D). Microbial phenotypes were predicted by bugbase (E).

The commensal microbial communities of crocodile lizards were significantly different from the surrounding environment, with these different habitats explaining 39.7% of the variation in microbial community structure (Figure 1(A)). The α-diversity analysis showed no significant differences in species richness (ACE index) among organs but significant differences in community diversity (Shannon index) among organs, with oral cavity having the lowest diversity (Figure 1(C)). Consistently, the results of β diversity analysis also showed that the bacterial communities clustered according to organs (Figure 1(A), p = 0.001). However, the PCoA result showed that the skin bacteria community did not cluster by skin sites (Figure 1(B)). Although PERMANOVA test revealed significant heterogeneity in the skin microbiota of the crocodile lizard (*p =* 0.001 in PERMANOVA test), this significant difference was attributed to differences in composition within groups rather than among skin sites (*p* = 0.001 in PERMDISP test). This suggests that the diversity of skin microbial community varies less among skin sites than among individuals.

In terms of community composition, more than 85% of the community was composed of Proteobacteria, Firmicutes, Bacteroidetes, Actinobacteria, and Acidobacteria, but abundances of the dominant bacteria differed among organs, and the skin microbial composition was similar across different skin sites (Figure 1(D)). In both the skin and gut, Proteobacteria was the most dominant phylum (>40%). In the oral cavity, Firmicutes (36.76%), Proteobacteria (27.16%), and Bacteroidota (24.30%) were the dominant phyla with relatively uniform abundance. Statistical comparisons among groups showed that Fusobacteriota was significantly enriched in the gut, Bacteroidota and Firmicutes were significantly enriched in the oral cavity; Deinococcota and Actinobacteriota were significantly enriched in the skin (Supplemental Figure S3A). This difference is also evident at the genus level: the skin was enriched with *Sphingomonas, Deinococcus, Novosphingobium, Nocardioides*, and *Spirosoma*, while the gut was enriched with *Plesiomonas, Stenoxybacter, Ligilactobacillus, Pseudomonas, Escherichia_Shigella*, and *Proteiniphilum* (Supplemental Figure S3B). Unlike the high diversity of the gut and skin microbiota, the oral bacterial community was mainly composed of *Mycoplasma* and *Filobacterium*, with a total abundance of 41.37% (Supplemental Figure S3B). In fact, the oral bacterial community was mainly composed of three strains, ASV988_*Mycoplasma iguanae*, ASV1234_*Mycoplasma insons*, and ASV2683_*Filobacterium* sp. These three ASVs were consistently detected in all oral samples but were almost undetectable in other organs and the environment (Supplemental Figure S4). Therefore, they were the core and signature species in the oral microbial community.

Microbial phenotypes in different organs were also analyzed. Aerobic bacteria were significantly enriched in the skin, anaerobic bacteria were significantly enriched in the gut and the oral cavity, while the abundance of facultative anaerobic bacteria did not differ significantly among organs (Figure 1(E)).

### Bacterial community assembly processes

**Figure 2.**
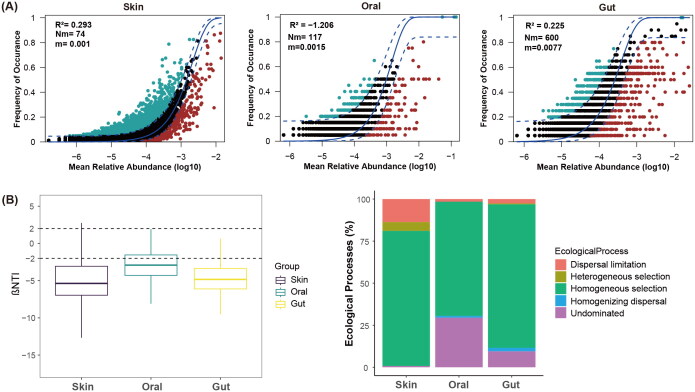
Bacterial community assembly processes. (A) The fit of the neutral community model (NCM) in skin, oral, and gut microbiota. The dashed lines indicate 95% confidence intervals of the NCM models, whereas the solid lines represent the best fit to the NCM model. Blue and red dots indicate the ASVs occurring more or less frequently than predicted from the models, respectively. R^2^ indicates the overall fit to the model, Nm indicates community size times dispersal rate, and m indicates dispersal rate. (B) The relative roles of ecological processes in governing the skin, oral, and gut bacterial communities were quantified using null model analysis. βNTI, beta nearest taxon index. |βNTI|>2 suggests deterministic processes, and |βNTI|<2 suggests stochastic processes.

Overall, the neutral community model was not a good predictor of the relationship between mean relative abundance and the frequency of ASV in body habitats (Figure 2(A)). Furthermore, the roles of deterministic processes (homogeneous and heterogeneous selection) and stochastic processes (dispersal limitation, homogenizing dispersal, and undominated) in governing the skin, oral, and gut bacterial communities were quantified. Skin, oral and gut bacterial communities were all primarily governed by deterministic processes (Figure 2(B), βNTI< −2). Homogeneous selection was the most important process in governing skin (80.28%), oral (67.89%) and gut (85.26%) microbial communities (Figure 2(B)), although the proportions of each ecological process vary between the wild and captive lizards (Supplemental Figure S5).

### Changes of skin microbiota over time

**Figure 3.**
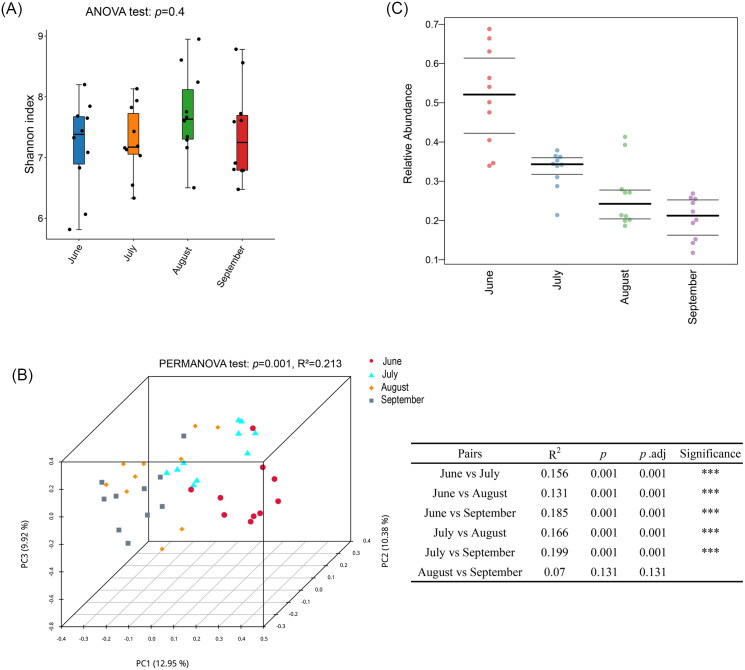
Temporal changes in the diversity and pathogenicity of skin microbiota. Alpha diversity was shown using Shannon indices (a). Differences in alpha diversity among groups were analyzed by ANOVA test. Beta diversity was displayed using a PCoA plot based on the Bray-Curtis distance matrix (B). Numbers in the parenthesis on the axis labels indicate the percentage of variation explained by each PC. Differences in beta diversity among groups were detected by PERMANOVA. Pathogenicity was shown using the abundance of pathogenic potential bacteria (C).

The skin microbiota (dorsal and ventral) of captive crocodile lizards was continuously monitored for 4 months in the Guangxi Daguishan Crocodile Lizard National Nature Reserve. There was no significant difference in alpha diversity of skin bacterial communities within four months (Figure 3(A), p = 0.4). For β diversity, PERMANOVA analysis showed that the skin microbial community was heterogeneous across time, with time accounting for 21.3% of the variation in skin microbiota (Figure 3(B), p = 0.001). Further multiple comparisons revealed no significant difference in β diversity between August and September, while significant differences were observed among other months (Figure 3(B)). Our results indicated that the β diversity of skin bacterial community fluctuated to a certain extent across time, especially between summer and autumn.

The prediction of biological phenotypes showed that the abundance of potentially pathogenic skin bacteria decreased gradually from June to September (Figure 3(C)), which was consistent with the higher incidence of skin diseases in crocodile lizards in summer and the lower incidence in autumn and winter.

### Effects of ex-situ conservation on commensal microorganisms

**Figure 4.**
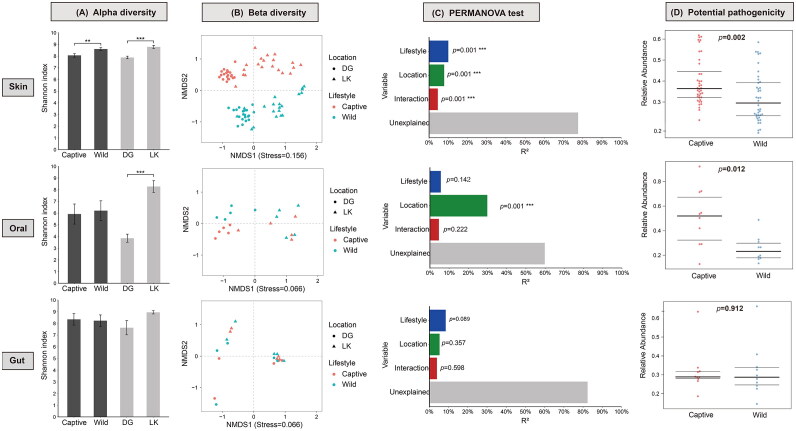
Diversity and pathogenicity of the bacterial community of crocodile lizard. Alpha diversity was shown using Shannon index (a), and differences between groups were compared by t-test. Error bars indicate the standard deviation. Beta diversity was showed using NMDS analysis based on binary Jaccard distance array (B). Differences in beta diversity between groups were detected by two-factor PERMANOVA (C), and percentage of variable explained (R^2^) and *p*-value were shown with significant effects marked with asterisk (*p* < 0.05). *p < 0.05; **p < 0.01; ***p < 0.001. Lifestyle categories includes wild and captive groups; location categories includes Luokeng and Dagui Mountain Nature Reserve groups. Pathogenicity was shown in abundance of potential pathogenic bacteria, and FDR-corrected p-values were generated by Wilcoxon test (D).

Both α and β diversity indicators showed significant differences in the diversity of skin bacterial microbiota between wild and captive crocodile lizards (Figure 4(A and B)). Captivity and location explained similar proportions of the variation in skin microbial diversity, 10.0% and 7.9%, respectively (Figure 4(C)). However, there was no significant difference in the diversity of gut and oral bacterial communities between the captive and the wild groups (Figure 4). Since the effect of interaction between location and lifestyle variables was significant in β diversity analysis (Figure 4(C)), the skin microbial data from two Nature Reserves were reanalyzed separately. The results showed that captivity did have a significant effect on the diversity of skin bacterial communities (*p* = 0.001 in both nature reserves).

Moreover, predictions of biological phenotypes revealed a significant increase in the potential pathogenicity of the skin and oral microbiota in the captive crocodile lizards, but no significant difference in the pathogenicity of gut microbiota between the captive and the wild groups (Figure 4(D)). Predictions based on the KEGG database also confirm this result (Supplemental Figure S6). Additionally, compared to the wild group, the skin microbiota of captive group had a significantly lower proportion of biofilm forming bacteria (*p* < 0.001), while no significant difference was observed in the gut and oral microbiota (Figure S7).

**Figure 5.**
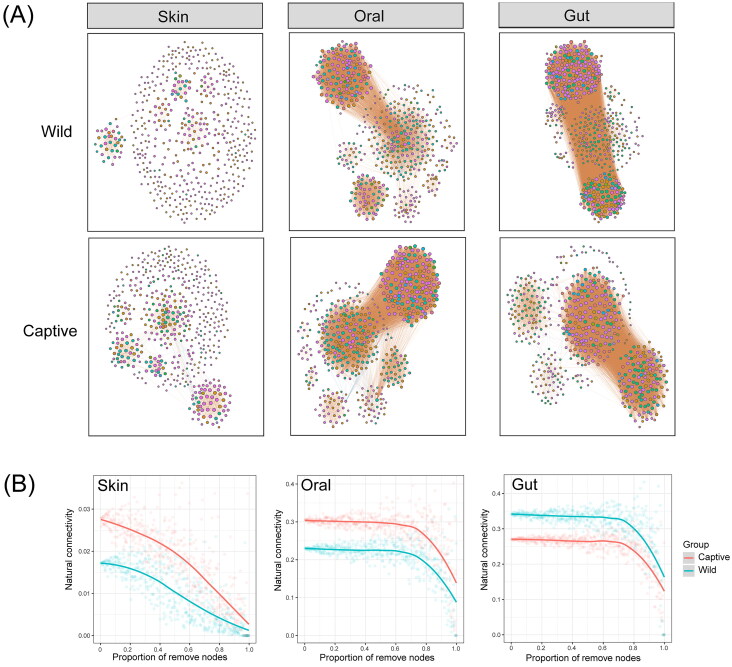
Co-occurrence networks and stability analysis for crocodile lizard symbiotic bacterial communities. (A) Networks were constructed based on the top 500 dominant bacterial ASVs. Nodes represent individual ASVs whose size are positively correlated with the node degree; edges represent significant Spearman correlations (*r* > 0.75 and *p* < 0.05), which red lines indicate as positive correlations, and blue lines indicate as negative correlations. (B) Stability analysis is shown as the relationships between bacterial natural connectivity and the proportion of removed nodes, such that larger shifts upon the same proportion indicate that there are less stability within bacterial networks.

Co-occurrence network analysis showed that bacterial networks of the gut and oral cavity were more complex than those of the skin (Figure 5(A)). Resistance of bacterial networks to disturbances was also tested (Figure 5(B)). In the gut and oral cavity, the natural connectivity of bacterial networks decreased only after 70% of nodes were attacked but remained stable before that point. In contrast, the natural connectivity of the skin bacterial network declined rapidly as the species were removed (Figure 5(B)). This suggests that bacterial networks of the gut and oral cavity are resistant to minor disturbances while the skin bacterial network is sensitive to disturbance. Moreover, the natural connectivity of the skin bacterial network decreased faster in the captive group compared to the wild group, indicating that the skin bacterial network in the captive group tended to be less stable. The trends in the natural connectivity of oral and gut bacterial networks in the captive group were the same as in the wild group (Figure 5(B)).

In addition, based on the analysis of different skin bacteria between the wild and captive crocodile lizards, the abundance of Actinomyces in the captive population was significantly lower than that in the wild population. At the genus level, the results of LEFSE, ANOVA, Stamp, and Wilcox statistics showed that the abundance of *Nocardioides* and *Sphingomonas* was significantly reduced in the skin of the captive group, while the abundance of *Deinococcus*, *Novosphingobium,* and *Fibrella* was significantly increased (Supplemental Figure S8). The abundance of *Novosphingobium* did not differ significantly between the wild and captive groups when data on the skin microbiota collected in different months were also taken into account.

### Relationship between microbiota and environment

The analysis of diversity showed that location have significant effects on the diversity of skin and oral microbiota other than gut microbiota (Figure 3).

**Figure 6.**
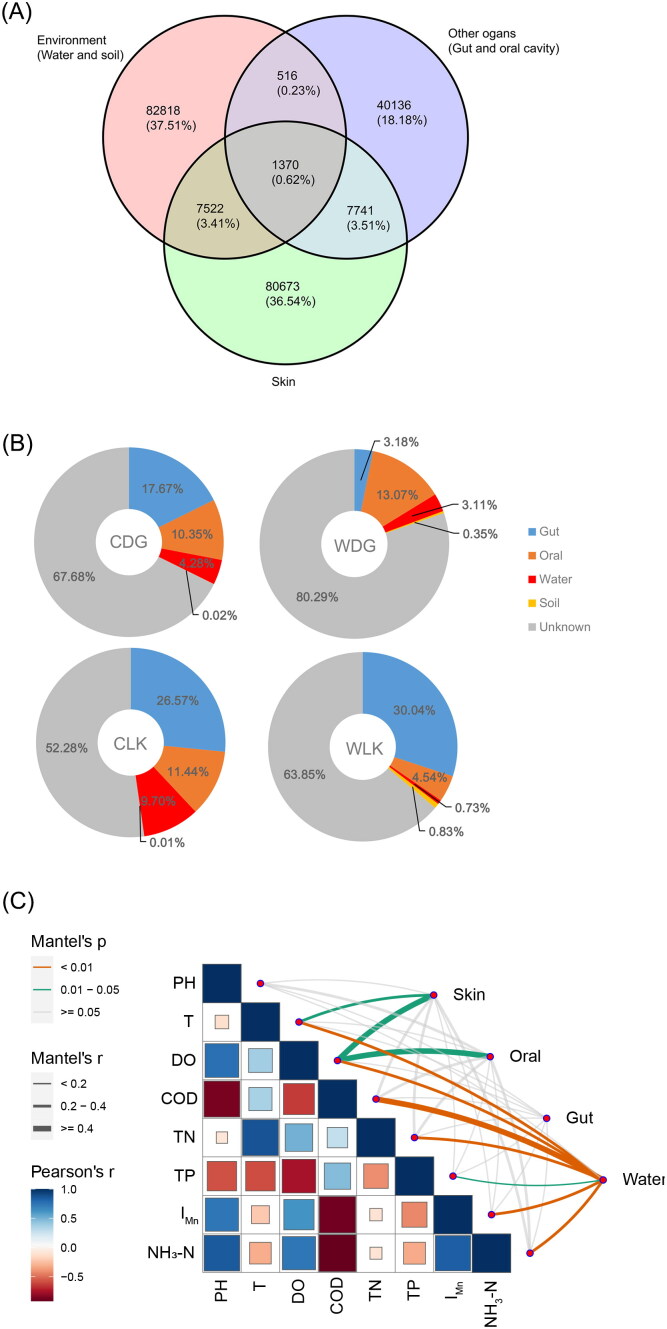
Relationship between skin micorbiota and other microbial communities. Overlapping ASVs among habitats were shown by a Venn graph (a). Source tracking of skin microbes in crocodile lizards was depicted with pie charts (B). CDG and WDG represent captive and wild crocodile lizards in the Dagui Mountain Nature Reserve, respectively; CLK and WLK represent the captive and wild crocodile lizards in the Luokeng Nature Reserve. The correlation between water quality indicators and microbial variation was assessed using Mantel and Pearson tests (C). Squares present the Pearson correlations between pairs of water quality indicator, such as pH, water temperature (T), dissolved oxygen (DO), chemical oxygen demand (COD), total nitrogen (TN), total phosphorus (TP), permanganate index (I_Mn_), and ammonia nitrogen (NH_3_–N). Lines indicate correlations between water quality indicators and microbial variation from skin, oral, gut and water. Line thickness indicates the r value, while color indicates the *p*-value in the Mantel test.

Among the 220,776 ASVs obtained in this study, the environment harbored the highest proportion of unique ASVs (37.51%), followed by the skin (36.54%). Of all the organs, skin shared far more ASVs with the environment than other organs did (Figure 6(A)). Most of the ASVs in the skin were unique to the skin, with only a few shared with other habitats. Among these, the skin shared slightly fewer ASVs with the environment than it did with other organs (Figure 6(A)).

According to the source of skin microbiota, the proportion of bacteria derived from the gut and mouth was much higher than that of bacteria derived from the environment (water and soil) although the proportion of each source differed among groups (Figure 6(B)). The environmentally-derived bacteria were mainly derived from water, with only a small proportion from the soil where the crocodile lizard resided. Therefore, we further examined the relationship between water quality indexes and microbial community changes. The results of correlation analysis showed that water quality had a weak effect on the skin and oral microbiota. Only water temperature (T) and dissolved oxygen (DO) were weakly correlated with changes of the skin microbial community (Figure 6(C)). There was no correlation between the variation of gut microbiota and the water quality indicators of habitat. Conversely, the changes in the water microbial community were greatly affected by water quality indicators, with 7 out of 8 indexes showing a significant correlation (Figure 6(C)).

## Discussion

In this study, 192 samples were collected from 6 distinct body sites of the crocodile lizards (head, back, belly and paw, oral cavity, and gut) and their habitats (water and soil), covering the main distribution area. To our knowledge, this is the first comprehensive study of the effects of ex-situ conservation on endangered animals.

### Niche specialization within the body-wide reptile microbiota resulted in different community traits and was adapted to organ function

In studies of humans and avians, body niche specialization leads to different microbial community characteristics (van Veelen et al. 2017; She et al. 2024; Zhu et al. 2024). Our findings revealed that the gut, oral, and skin habitats recruited distinct microbiota and specific biomarker taxa could differentiate body habitats, suggesting niche specialization in the reptile as well. However, within the same organ, the differences in the skin microbiome between skin sites were less pronounced than those between individuals.

The skin recruited dominant aerobic bacteria and UV-resistant bacteria (*Sphingomonas* and *Deinococcus* (Gerber et al. 2015; Harel et al. 2023)), while the gut and oral cavity accumulated more anaerobic bacteria, which could be attributed to skin’s constant exposure to air and UV stress. The predominance of aerobic respiration in the skin has also been reported in human microbiome (She et al. 2024). In line with its absorption function, the intestine recruited more *Proteiniphilum*, which has the ability to hydrolyze carbohydrates and proteins (Orellana et al. 2022). We identified *M. iguanae*, *M. insons*, and *Filobacterium* sp. as the core and signature species of the oral microbiota, because they were present in high abundance in every oral sample and were almost undetectable in other organ and environmental samples. *Mycoplasma iguanae* was isolated from spinal disease in feral iguanas (*Iguana iguana*), but a subsequent experimental inoculation did not induce lesions (Brown et al. 2006; 2007). *Mycoplasma insons* was cultured in high numbers from the posterior choanae and upper tracheae of healthy green iguanas, and was considered as normal flora in the respiratory tract of iguanas (Brown et al. 2007). Overall, this study provides evidence of the association of microbial taxa with the key physiological functions of organs.

Although the symbiotic microorganisms of reptiles exhibited niche specificity as do other vertebrates, the dominant bacteria in each organ of reptiles differed from those in other vertebrates. This is understandable given that host phylogeny is a significant factor influencing the differences in vertebrate-associated microbial communities across species (Huang et al. 2022, Ross et al. 2018).

### The bacterial community in lizard assembly was mainly shaped by deterministic processes

Microbial community assembly is essential to understand the mechanisms by which microbiome regulate ecosystem functions (Luan et al. 2020). The dominance of homogeneous selection in microbial assembly indicated that bacterial colonization on the skin, oral cavity, and gut of the crocodile lizard was primarily a result of host selection rather than random interactions with environment. In Chelonia, homogeneous selection was also found to be the most critical process in governing oral and skin microbial communities of Chinese softshell turtle in the rice-fish culture system (Ding et al. 2022). This is different from bird, where the neutral model could explain 40.4% of variation in gut, 55.9% in mouth, and 45.2% in skin microbiota, respectively (Zhu et al. 2024). In Chelonia, the proportion of deterministic and stochastic processes varied under different aquaculture systems (Ding et al. 2022). However, in our study, while the proportions of each ecological process varied between the wild and captive lizards, deterministic processes consistently dominated. Therefore, the taxonomic status of the host has an important influence on the assembly of its symbiotic microbiome.

### Effects of ex situ conservation on reptile bacterial community were mainly manifested in skin microbiota

In terms of the effects of ex-situ conservation on the animal microbiome, most current studies have focused on the gut microbiome, with only a few addressing the skin microbiome, and none exploring the oral microbiome, let alone a systematic assessment of microbial communities across different organs.

Previous studies on gut microbiota showed that alpha diversity generally decreases in captive animals with varying degrees of decline among species (Dallas and Warne 2023). In this study, no significant difference was detected in the alpha and beta diversity of gut bacterial communities between captive and wild crocodile lizards or between different locations. A previous investigation into crocodile lizard gut microbiota in a single nature reserve also revealed no significant difference in alpha diversity (Simpson index) between wild and captive groups, but significant differences in beta diversity (Tang et al. 2020). Examination of the scatter plot of beta diversity revealed individual heterogeneity in gut microbiota, with no clustering observed based on lifestyle or location. This underscores the importance of extensive sampling. The lack of significant differences in gut microbiota between wild and captive populations and across different locations may be attributed to dietary factors. Because diet and phylogeny are the most significantly associated with gut microbiome diversity across reptile and other vertebrate (Huang et al. 2022; Hoffbeck et al. 2023). Currently, the diet of captive crocodile lizards in various nature reserves is consistent, primarily comprising earthworms supplemented with other trapped insects, which mirrors the food residues found in the gut of wild populations (Tang et al. 2020). Coupled with the stable oral microbiota observed in both captive and wild populations, this suggested that the current diet used in ex-situ conservation efforts for crocodile lizard was appropriate.

In contrast to the relative homogeneity observed in gut microbial community, this study revealed significant alterations in the skin microbiota of the captive crocodile lizard, characterized by a notable decrease in α diversity and significant differences in β diversity compared to the wild population. Those changes in the skin microbiota were evident across two distinct nature reserves, indicating that they were not accidental. The skin microbiota plays a crucial role in maintaining skin barrier and has been implicated in the protection against infection by pathogenic microbes (Harris-Tryon and Grice 2022). Accumulated evidence from previous studies on mammals, fish, reptiles, and amphibians generally indicates that healthy skin harbors a diverse skin microbiota, and that dysbiosis of skin microbiota is associated with disease (Lauer et al. 2008; Becker and Harris 2010; Allender et al. 2018; Ross et al. 2019; Pardo et al. 2024). Furthermore, the dynamic change in skin microbiota is disease severity-dependent and could normalize gradually during treatment in some diseases (Jani and Briggs 2014; Naik et al. 2020; Khadka et al. 2021). Our previous studies on crocodile lizard showed that dermatosis was significantly associated with changes in skin microbiota rather than gut microbiota (Jiang et al. 2022, 2017). The significantly lower diversity of skin microbiota observed in captive crocodile lizards may suggest an increased susceptibility to skin diseases.

Notably, the captive crocodile lizards had a higher abundance of potentially pathogenic bacteria in their skin compared to those in the wild. In addition, the abundance of potentially pathogenic bacteria in the skin decreased from June to September. This finding aligns with the higher prevalence of skin diseases observed in captive crocodile lizards, particularly during summer compared to autumn and winter (Jiang et al. 2022). Although potential pathogens require specific conditions to cause disease, their enrichment increases the chance and risk of infection in captive animals, especially those are immunocompromised, where normal skin bacteria may become pathogenic (Ross et al. 2019). Similar enrichment of opportunistic pathogens were also found in the skin of captive giant pandas (Ma et al. 2021). In contrast, no such changes in potential virulence were observed in the gut microbiota. Furthermore, *Nocardioides* and *Sphingomonas*, whose abundance was significantly reduced in the skin of captive group, have known function in inhibiting bacterial or fungal pathogens (Kubota et al. 2003; Innerebner et al. 2011). The reduction of probiotics in the skin may thus be another side effect of captivity, leaving the host vulnerable to pathogens exposure.

Microorganisms have evolved to form biofilm, facilitating symbiotic relationship with hosts and enhancing survival in harsh environmental conditions (Rather et al. 2021). The significantly lower proportion of biofilm-forming bacteria in the skin microbiota of the captive group implied that the skin microbial community may be less stable compared to the wild group. Indeed, in the analysis of co-occurrence networks, we found that, compared with oral and intestinal microbial networks, skin microbial networks were simpler and exhibited greater instability, indicating that skin microbial networks were more sensitive to external attacks than oral and intestinal microbial networks. In line with this, the study on human microbiome has shown that the stool and oral microbiome are more stable than the skin and nasal microbiome, and individuals with disease possess a less stable microbiome (Zhou et al. 2024). Similarly, a study on fish disease revealed that the microbial networks of diseased fish are simpler than those of healthy fish (Zhang et al. 2024). Additionally, the stability of the skin microbial network in the captive group was inferior to that observed in the wild group. The unstable network made the skin of the captive group more vulnerable when faced with the infections from pathogens or environmental stress. This phenomenon has also been observed in pandas, where the microbial networks of captive pandas are less complex and less stable than those of wild pandas (Cui et al. 2023).

In addition, the microplastic contamination, generated by human activities in ex situ conservation, is also one of the side effects of captivity on skin microbiome. Our previous investigations have shown that the crocodile lizards and their habitat are experiencing considerable microplastic pollution, which carries pathogenic bacteria and transfers them to the skin of crocodile lizards (Xie et al. 2023, 2024).

Collectively, the effects of captivity on commensal bacterial community of reptile were predominantly manifested in skin microbiota. It can be inferred that alterations in the skin microbiota in captive crocodile lizards were associated with an increased prevalence of skin diseases in this population. Our study also highlighted the importance of simultaneously profiling microbial communities across multiple organs to obtain a more comprehensive understanding of the animal microbiome when evaluating the effects of captivity. In the future, the side effects of captivity could be reduced by manipulating the skin microbiota, such as probiotic addition or microbiota transplantation, thereby improving the likelihood of survival in reintroduction programs.

### Importance of environment in reptile skin microbiota variation

Given that reptiles move in close contact with their environment, we investigated whether changes in skin microbiota were entirely caused by the environmental factors. As a barrier between the body and the environment, the skin did share more microorganisms with the environment than any other organ. However, most of skin microbes were endemic, with only a small proportion being identical to environmental microbes, even less so than those shared with other organs.

On a large scale, location significantly impacted skin microbiota, which was consistent with previous findings in other animal groups (Ross et al. 2018). However, only 7.9% of the variation in skin microbiota could be explained by location, a smaller proportion than that explained by captivity. On a small scale, source tracking within populations showed that most skin microbes did not derive from their habitat surroundings-water and soil. In contrast, there were more skin microbes from the gut and mouth than from environment. This indicated that the migration of microbiota between different organs was greater than that of the environment and skin. This result aligns with recent study on symbiotic microbes of crested ibis (Zhu et al. 2024), contrasting with previous studies of captive Komodo dragons and two sympatric birds, which suggested a substantial environmental contribution to skin microbial variation (Hyde et al. 2016; van Veelen et al. 2017).

A limitation of our study is the relatively small number of environmental sources, resulting in a large proportion of skin microorganisms with unknown origin. Unexpectedly, for semi-aquatic crocodile lizards, the effect of water on variation in the skin microbiota was weak. This explains why the ventral skin, in contact with ground, exhibited no significant difference in microbiota compared to dorsal skin.

Collectively, our results indicated that skin microbiota of reptile operates as a relatively independent system, and the effects of captivity on skin microbiota were not entirely caused by environmental changes. The dominance of homogeneous selection of microbial assembly also supported this result.

It can be predicted that, without artificial regulation, the skin microbial community of the captive reptile will partially recover through time after being reintroduced to the wild, but it is difficult to fully recover to the original wild state. This phenomenon has been observed in a soft-release of amphibian *Atelopus varius,* and their skin microbiota also increased antifungal function (Kueneman et al. 2022). According to the results of this study, “rewilding the skin microbiota” can be achieved through a combination of artificial regulation of skin microbiota and pre-release to the wild, thereby improving the survival rate of endangered reptiles.

## Conclusion

We have generated a comprehensive biogeo-graphical microbial mapping of surface organs of the endangered crocodile lizard. The symbiotic microbiota of the crocodile lizard significantly differed from habitat in both species composition and diversity. While significant differences were found in microbial community diversity among organs, no significant differences were found in species richness. More than 85% of the bacterial communities were composed of Proteobacteria, Firmicutes, Bacteroidota, Actinobacteria and Acidobacteria. But the skin microbiota did not exhibit clustering according to specific skin sites. The oral microbiota exhibited the lowest diversity, with the core microbiome dominated by *Mycoplasma* and *Filobacterium.* More important, this study showed that the influence of ex situ conservation on endangered crocodile lizards was primarily manifested in the skin microbiota other than the gut microbiota or oral microbiota. Our findings contributed to a deeper understanding of reptile-associated microbes across various organs and their habitats. More importantly, they provided insights into the effects of ex situ conservation and suggest future directions for improvement. While ex situ conservation offers clear benefits for endangered animals, this study underscored the need to carefully consider and mitigate potential adverse effects. Moreover, for reptiles, increasing *in situ* conservation efforts are crucial to mitigate the impacts of environmental changes and captivity-related factors. In the case of crocodile lizards, based on current conservation achievements, future ex situ efforts should prioritize the preservation of skin microbiota over gut and oral microbiota as captivity reduces skin microbial diversity and stability.

## Supplementary Material

Supplemental materials.doc

## Data Availability

The raw sequence data reported in this paper have been deposited in the Genome Sequence Archive in National Genomics Data Center, China National Center for Bioinformation/Beijing Institute of Genomics, Chinese Academy of Sciences (GSA: CRA016506, CRA 016511, and CRA 016512) that can be publicly accessible at https://ngdc.cncb.ac.cn/gsa. The associated BioProject number was PRJCA026109.
