# Supplementary material for: Effects of ex situ conservation on commensal bacteria of crocodile lizard and conservation implications
Source: Vet Q. 2025 Feb 10;45(1):1–14. doi: 10.1080/01652176.2025.2463704 (PMC11816626; doi:10.1080/01652176.2025.2463704)
Supplement: Supplemental materials.doc [file TVEQ_A_2463704_SM6130.doc]

**Supplemental Materials**

**Effects of ex** **situ conservation on commensal bacteria of crocodile lizard and conservation implications**

Haiying JIANG^1#^, Mei LV^1#^, Tengfei HE^1^, Mujiao XIE^1^, Zhiwen ZHAO^1^, Jiasong HE^2^, Shuyi LUO^2^, Yide GUO^1^, and Jinping CHEN^1*^

^1^ Guangdong Key Laboratory of Animal Conservation and Resource Utilization, Institute of Zoology, Guangdong Academy of Sciences, Guangzhou 510260, China

^2^ Guangxi Daguishan Crocodile Lizard National Nature Reserve, Hezhou 542800, China

^*^*Correspondence*: 105 Xingang West Road, Haizhu District, Guangzhou 510260, China. E-mail: chenjp@giz.gd.cn

^#^ Authors contribute equally to this study.

**Table S1 Search results for articles on non-human vertebrate microbiome utilizing the Web of Science database**

| Search mode | No. of articles | Percent |
| --- | --- | --- |
| (TS=(vertebrate microbiome)) NOT TS=(human) | 11809 | 100% |
| (TS=(vertebrate microbiome)) NOT TS=(human) AND TS=(gut) | 9176 | 77.70% |
| ((TS=(vertebrate microbiome)) NOT TS=(human)) AND TS=(oral) | 1018 | 8.60% |
| ((TS=(vertebrate microbiome)) NOT TS=(human)) AND TS=(skin) | 521 | 4.40% |


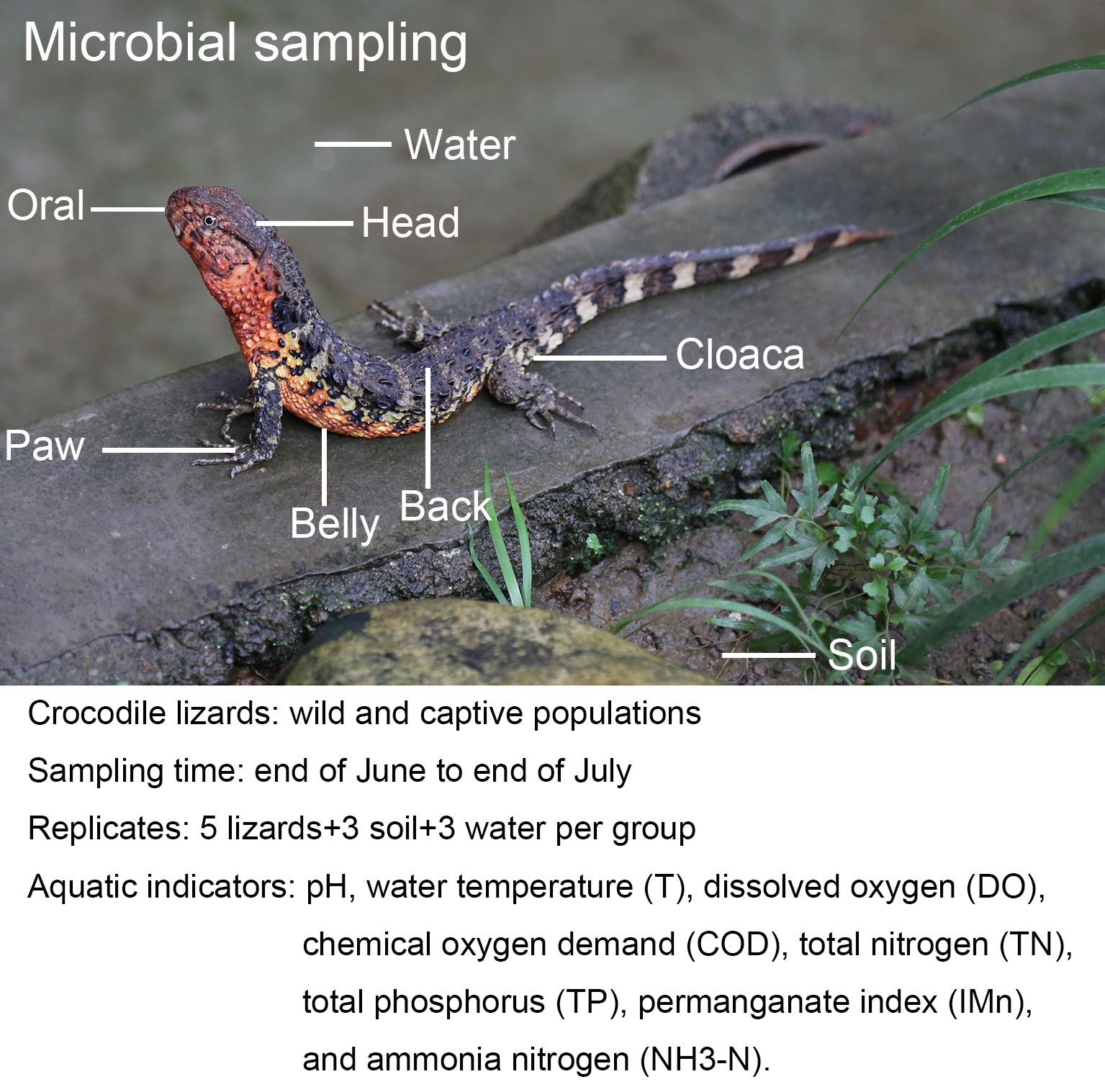


**Figure S1 Microbiome profiling in crocodile lizards and habitat.**

**
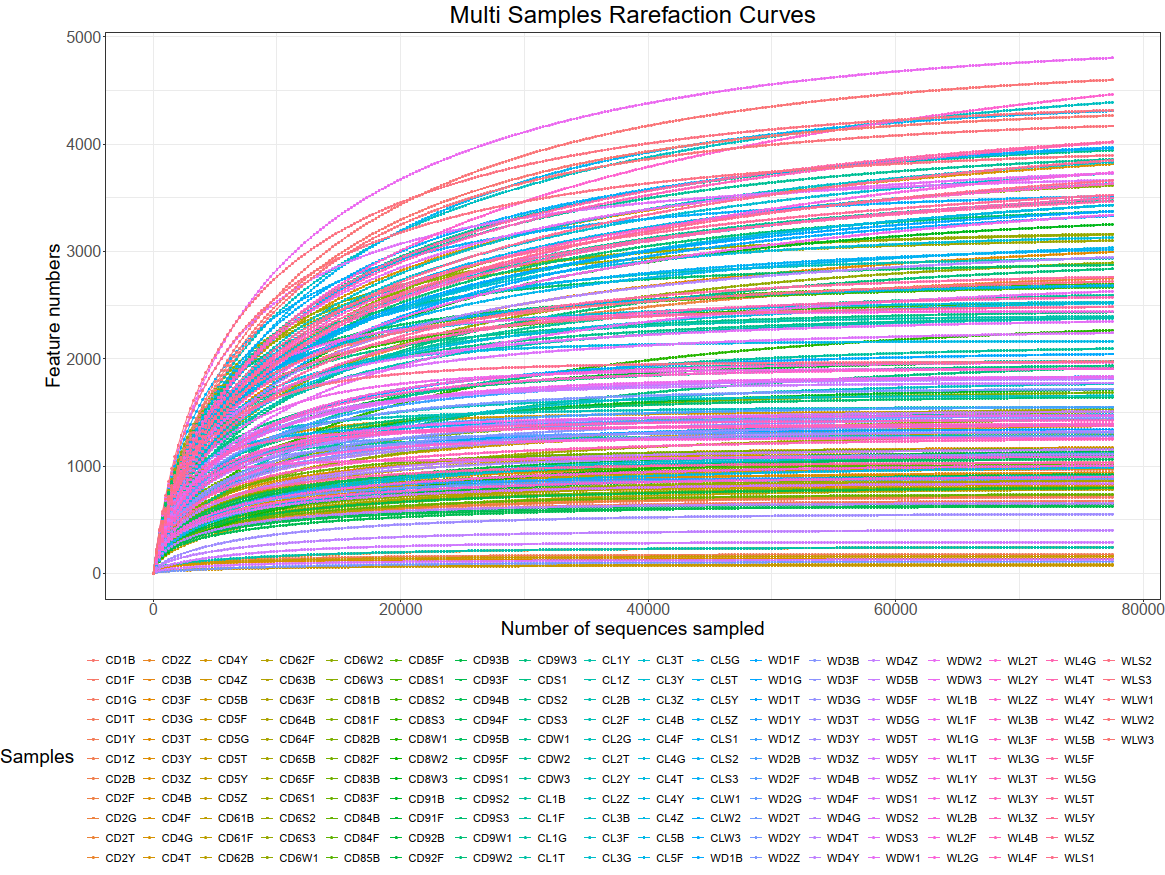
**

**Figure S2 Rarefaction curves.**

**
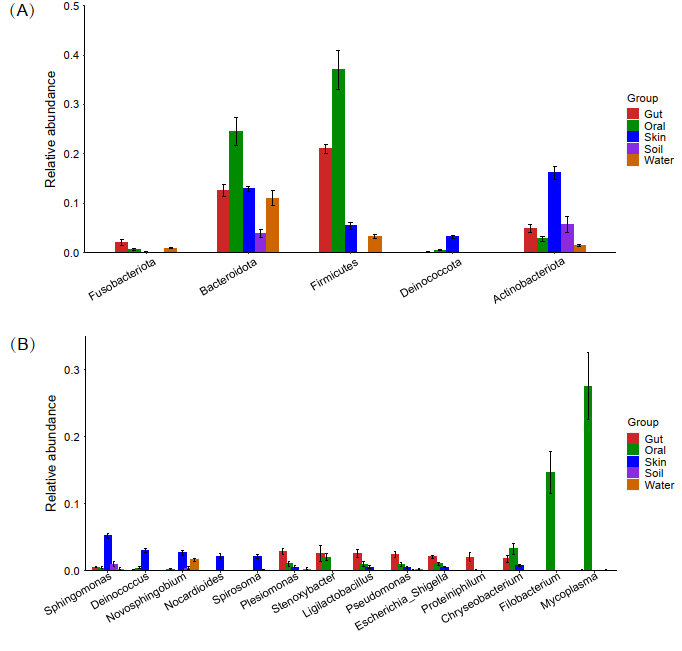
**

**Figure S3 Relative abundance of bacteria displaying significant differences across groups at Phyla (A) and genus (B) levels.** **Bacteria** were screened using the LEfSe method with LDA>4.0.


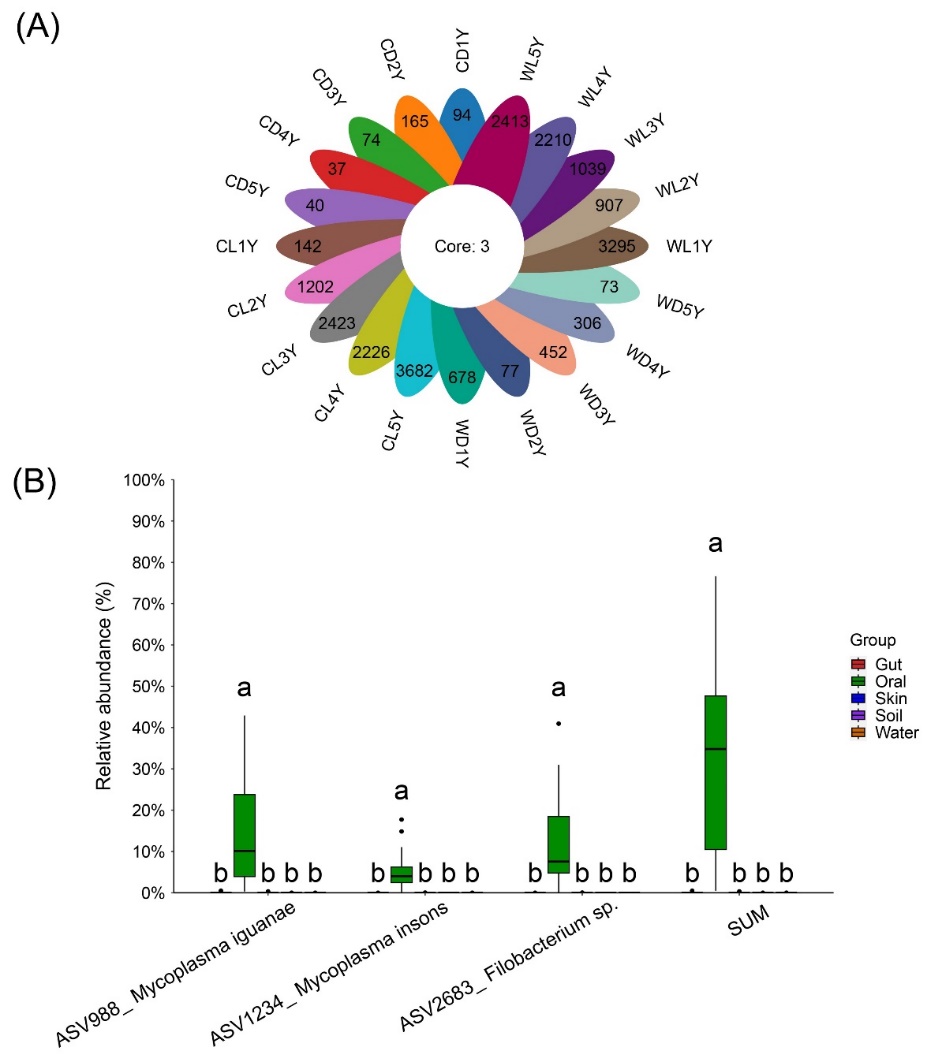


**Figure S4 Core bacteria of the oral microbiota of crocodile lizards.** The number of common bacteria among samples was showed in a Venn diagram (A). Abundances of these common bacteria and their total abundance were shown in boxplots (B). Data with different letters above the bars are significantly different (*p*<0.05) determined by ANOVA test and Scheffe’ multiple comparison.

**Figure S5 Relative roles of ecological processes in governing the skin, oral, and gut bacterial communities were quantified using null model analysis.**


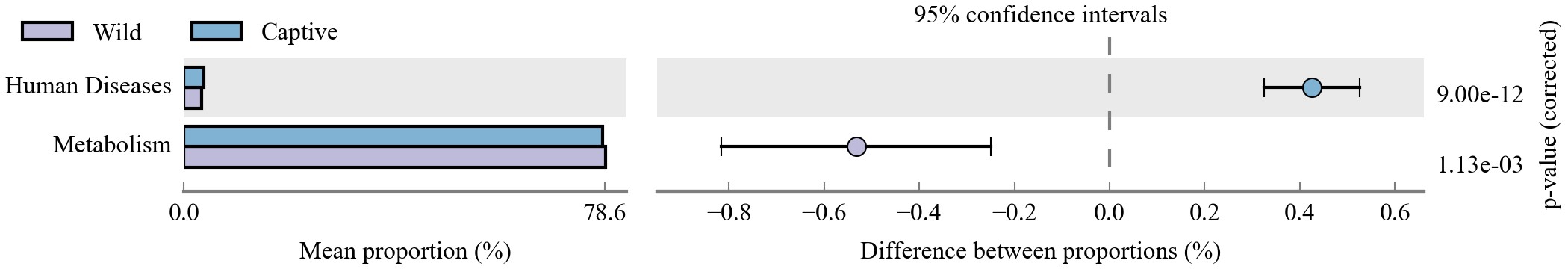


**Figure S6 Skin bacterial function with significant differences between wild and captive groups.** Level1 prediction based on KEGG database was conducted by PICRUST2 software. There were no significant differences in the function of the gut and oral microbial communities between the wild and captive groups.


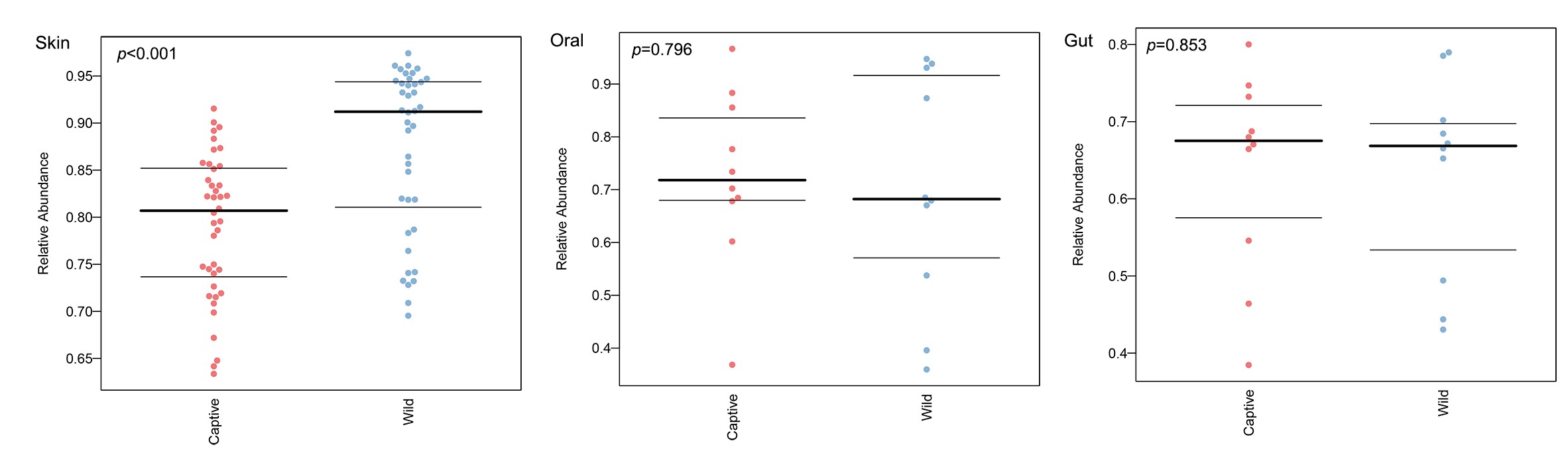


**Figure S7 Abundance of biofilm-forming bacteria in the crocodile lizard symbiotic microbial communities**. FDR-corrected *p*-values were generated by Wilcoxon test.


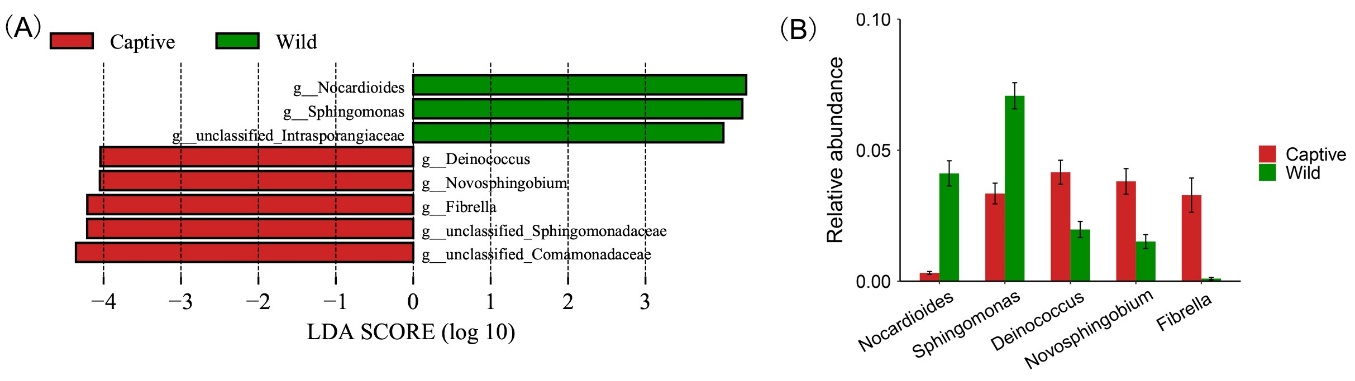


**Figure S8** **Skin bacteria with significant differences in abundance between captive and wild crocodile lizards were screened using LEfSe analysis, ANOVA, Stamp and Wilcox statistics (*p*<0.05).** (A) Comparison results from LEfSe analysis, LDA>4. (B) Relative abundances of the screened bacteria. Error bars represent standard error (SE).
